# An analysis of patient motivation for seeking online treatment for binge eating disorder—A mixed methods study combining systematic text condensation with sentiment analysis

**DOI:** 10.3389/fpsyt.2022.969115

**Published:** 2022-11-04

**Authors:** Trine Theresa Holmberg, Maxime Sainte-Marie, Esben Kjems Jensen, Jakob Linnet, Eik Runge, Mia Beck Lichtenstein, Kristine Tarp

**Affiliations:** ^1^Research Unit for Digital Psychiatry, Center for Digital Psychiatry, Mental Health Services in the Region of Southern Denmark, Odense, Denmark; ^2^Department of Political Science, Danish Center for Studies in Research and Research Policy, Aarhus University, Aarhus, Denmark; ^3^Department of Clinical Research, Faculty of Health Sciences, University of Southern Denmark, Odense, Denmark; ^4^Clinic on Gambling- and Binge Eating Disorder, Department of Occupational and Environmental Medicine, Odense University Hospital, Odense, Denmark

**Keywords:** binge eating disorder (BED), internet based intervention, self-determination theory (SDT), mixed method approach, sentiment analysis, cognitive behavioral therapy (CBT)

## Abstract

**Objective:**

Online treatment for binge eating disorder (BED) is an easily available option for treatment compared to most standard treatment procedures. However, little is known about how motivation types characterize this population and how these impact treatment adherence and effect in an online setting. Therefore, we aimed to investigate a sample of written motivation statements from BED patients, to learn more about how treatment and online treatment in particular, presents in this population.

**Methods:**

Using self-determination theory in a mixed methods context, we investigated which types of motivation were prevalent in our sample, how this was connected with patient sentiment, and how these constructs influence treatment and adherence.

**Results:**

Contrary to what most current literature suggests, we found that in our sample (*n* = 148), motivation type was not connected with treatment outcome. We did find a strong association between sentiment scores and motivation types, indicating the model is apt at detecting effects. We found that when comparing an adult and young adult population, they did not differ in motivation type and the treatment was equally effective in young adults and adults. In the sentiment scores there was a difference between sentiment score and adherence in the young adult group, as the more positive the young adults were, the less likely they were to complete the program.

**Discussion:**

Because motivation type does not influence online treatment to the same degree as it would in face-to-face treatment it indicates that the typical barriers to treatment may be less crucial in an online setting. This should be considered during intake; as less motivated patients may be able to adhere better to online treatment, because the latter imposes fewer barriers of the kind that only strong motivation can overcome. The fact that motivation type and sentiment score of the written texts are strongly associated, indicate a potential for automated models to detect motivation based on sentiment.

## Introduction

### Binge eating disorder

Binge Eating Disorder (BED) is characterized by repeatedly experiencing loss of control on eating behavior and consuming large amounts of food within a short period of time, which is perceived as shameful and stressful (1). The severity of BED depends on the number of binge eating episodes per week. A distinguishing characteristic of this type of eating disorder (ED) is that the behavior is neither associated with restrictive eating nor compensatory behavior, such as vomiting or excessive exercise.

Although BED is not as well-known as anorexia nervosa (AN) and bulimia nervosa (BN), the occurrence of BED is higher (2). Prevalence is estimated to be 2–4% of the adult population in Europe and the U.S. (2–6). The average duration of BED is between 4 and 8 years (2, 3), while the gender distribution in BED is about one-third males and two-thirds females (7). It is estimated that about 80% of people with BED have some comorbidity (3), the most common being; Depression, Anxiety Disorders, Post Traumatic Stress Disorder, and Alcohol Use Disorder (8).

One theoretical model has suggested that individuals with BED use food to regulate emotions (9), defined as a dysfunctional attempt to influence which types of emotions are experienced (10). The theory of emotional eating suggests that overeating is a coping strategy for dealing with emotional stress (11). The avoidance theory suggests that overeating is a way to get rid of negative emotions while eating (12). Thus, the treatment approach focused on emotional triggers and teaching healthy strategies to cope with mental distress.

### Internet-based cognitive behavioral therapy for eating disorders

In later years, a growing body of evidence for the effectiveness of internet-based Cognitive Behavioral Therapy (iCBT) as treatment for EDs has emerged (13). Guided self-help is recommended in NICE guidelines as the first line intervention, on a stepped care model for BED (14).

Internet-based guided self-help is a specific type of iCBT where patients receive psychoeducation, cognitive behavioral exercises, and online feedback from a health professional. The communication is mainly written, usually asynchronous, and time-expenditure for the therapist is significantly lower than in face-to-face treatment as therapists in this program expended ~15 min a week as opposed to 1 h in treatment as usual.

A randomized controlled trial (RCT) on iCBT for BED, BN, and Eating Disorders Not Otherwise Specified (EDNOS) by ter Huurne et al. (15) found that female patients with BED reduced eating psychopathology over time. Another study by Wagner et al. (16) also found that iCBT was effective in providing long term reduction in ED pathology and binge episodes. Yet another study by Kindermann et al. (17) found that iCBT programs with individualized intensity of support may reduce ED-related symptoms study by de Zwaan et al. in the INTERBED non-inferiority RCT (18), demonstrated that face-to-face CBT had a faster effect and larger symptom reduction than iCBT at 6 months follow-up. However, the iCBT still demonstrated significant reductions in symptomology and the efficiency achieved through the iCBT program was stable over time. The authors concluded that iCBT is a viable, lower threshold treatment-type than regular CBT. In addition, iCBT with therapist support proved more effective than both waitlist and self-study (CD-ROM/bibliotherapy) (18).

### Motivation and the self-determination theory

According to Ryan and Deci's theoretical framework the self-determination theory, motivation can be controlled or autonomous: Controlled motivation is contingent on external factors such as punishment and reward, based on desire for approval or avoidance of shame. These factors are not integrated into the self, and people feel a pressure to conform to certain standards (19). When individuals are motivated autonomously, they have integrated both intrinsic and extrinsic motivational factors into their sense of self, which gives the individual a sense of volition (19). Intrinsic motivation is achieved when a task is meaningful and enjoyable. Extrinsic comes in several forms and can also be a potent type of motivation depending on the degree of integration. Extrinsic motivation is divided into four subgroups going from more controlled to more autonomous (20): (1) The most controlled form of extrinsic motivation is called external regulation, which is distinguished by external rewards, compliance and reactance. (2) Less controlled but still mainly external is introjection, which has some ego involvement, and this motivation type is mainly motivated by approval from self and others (20). (3) Moving toward more autonomous motivation, the next step is identification, which while still being extrinsic, has more conscious valuing of the activity and more personal endorsement. (4) The most autonomous of the extrinsic motivation types, is integration. This motivation type is categorized by a consistency in the internalization of the motivation, high agency in the individual and is just a step away from being intrinsic motivation (20). How internalized motivation is, connects with the perceived locus of causality. It depends on if the event occurring is perceived as caused externally or internally, or said in another way, if the person has integrated a sense of responsibility and agency (21).

### The importance of motivation in internet-based eating disorder treatment courses

In regards to EDs, shame and outward appearance are significant factors, and motivation is an important factor in recovery from EDs (22). According to an examination of the role of autonomous vs. controlled motivation in predicting inpatient treatment outcome for anorexia nervosa, autonomous motivation pretreatment was shown to improve treatment outcome, and this association was not found in controlled motivation (23). Another study on autonomous and controlled motivation for ED treatment (24) emphasized the importance of nurturing autonomous motivation and using autonomy supportive strategies in ED treatment to improve outcome.

Compared to regular face-to-face treatment, however, internal self-regulation or autonomous motivation may be even more critical when it comes to the patient's ability to complete a self-help program on the internet (25). Since the ability to self-regulate, according to self-determination theory, depends heavily on motivational factors, it is important to investigate the impact of motivation among people with BED receiving internet-based treatment. It is also of importance because we want to include the patients who can benefit the most and exclude the ones who do not benefit from our intervention. A failed intervention can be detrimental to future outreach for help, *via* a sense of defeat. Other treatment options should be recommended instead, or special care taken for this population, such as focus on motivational work before starting treatment. This can also be a way to optimize therapist resources for the population who needs them the most. To the authors' knowledge, no studies have previously explored this.

We divided the sample into young adults and adults to discern if there were any differences in motivation that should be considered during the treatment of BED online.

### Aims and objectives

The aims of the present study were 4-fold. The first objective was to describe the patient sample characteristics according to age, body mass index (BMI), binge eating, depression, and quality of life. The second objective was to qualitatively explore patient motivations for seeking BED treatment in general and online treatment specifically and uncover whether those motivations were of an autonomous and/or controlled character. The third objective was to statistically examine the effect of motivation on treatment completion and treatment effect. The fourth objective was to combine the qualitative findings with computerized sentiment analysis to investigate how written statements can be used clinically.

### Hypotheses

We hypothesized that the more integrated the motivation, the better adherence and/or treatment outcome.

We also hypothesized that there could be a correlation between sentiment of written patient motivational statements and motivation type.

## Methods

### Design

The research design of this study was mixed methods (26), more specifically the triangulation design, with data transformation of the qualitative data into quantitative, analyzing both types separately and in combination. This design was chosen because we wanted to examine if and how motivation type based on qualitative findings would influence treatment outcome and adherence. We also wanted to explore if the written statements correlates with sentiment polarity score, thereby gaining information from patient statements to inform the quantitative findings, in a manner that a pure qualitative or quantitative design alone could not.

We collected quantitative data from the patients at start and finish of their treatment. Qualitative data were collected at start of treatment, as written answers to two questions.

The qualitative data was transformed into quantitative data in two ways, manually, by systematic text condensation, and computerized by the Sentida package.

The questions, patients answered were:

“*What is the reason for your request? What do you require help with?”*

“*What made you choose internet based treatment instead of other treatment?”*

### Patients

Patients were adults (≥18) who had applied for internet-based BED treatment iBED, with a first come, first served policy. The program was advertised on the mental health services webpage, the head of the project (MBL) was on the news reporting on the program, and wrote two articles on videnskab.dk, which is a science dissemination site for non-academics.

Exclusion criteria was severe BED, MDI >40, moderate to severe comorbidity, such as personality disorders and schizophrenia.

Clinical psychologists assessed the applications and rated the BED symptoms and severity based on the in house questionnaire Binge Eating Disorder Questionnaire (BED-Q) and the Eating Disorder Examination Questionnaire (EDE-Q) (27).

We divided completion rate into three groups. Low (*n* = 54), high (*n* = 56) and full (*n* = 37). Low is patients who completed <6 session. High is patients who completed <10, and 10 plus follow-up were considered full.

We also divided the patients into adults and young adults for some tests, to discern if proposed brain immaturity could affect treatment effect (28).

### Treatment program

The iBED program was developed at the Center for Digital Psychiatry in the Mental Health Services in the Region of Southern Denmark. It contains an internet-based treatment intervention for mild to moderate BED with written support from a therapist. The iBED program is based on a cognitive behavioral approach, consisting of psychoeducation, goal setting, exploring eating patterns, behavior change, coping strategies, relapse prevention, and assessment of binge eating symptoms. The program entails ten sessions whose content is shown in Table 1. Before the first, between sessions, and after the last sessions, patients filled out questionnaires. The program was delivered by the digital platform Minddistrict (29) and is free of charge.

**Table 1:** The iBED program session content.

Session 0	Introduction to platform, psychoeducation, the BED diagnosis, CBT and iCBT
Session 1	Psychoeducation about physical and psychological consequences of BED, motivation for treatment, pros and cons of treatment. Exercise: The patient is asked to describe pros and cons of being in treatment
Session 2	Exercise: The patient is asked to make a list of goals in treatment
Session 3	Psychoeducation about the brain's reward mechanisms, feelings of hunger and satiety, and diets. Exercise: The patient is asked to write down food intake during the day for a week
Session 4	Psychoeducation about “all or nothing” thought pattern, benefits of eating regularly and a stabilized eating pattern. Exercise: The patient is asked to eat slowly and calmly while continuing the food journal
Session 5	Psychoeducation about the psychological courses of binge-eating and eating as a non-suitable way to handle emotions. Exercise: The patient is asked to register their binge-eating behavior in the journal: Which situations caused the binge-eating episode? Which thought they had, as they felt the urge to binge-eat? Which emotions and physical reactions they felt before the binge-eating episode? How and what they ate? How they felt afterwards?
Session 6	Psychoeducation about the connection between food and emotions, emotion regulation, and allowing thoughts. Exercises: To give advice to a fictional character who is about to have a binge eating episode. To find write down a list of activities and sentences, that can be alternatives to binge-eating behavior
Session 7	Exercise: Daily registration of the patients own evaluation of themselves and their way of avoiding binge-eating behavior
Session 8	Psychoeducation about kindness toward oneself in a process of change. Exercise: The patient is asked to write down caring and positive sentences about themselves, that they can use, when things get hard
Session 9	Prevention of relapse. Exercise: To state down factors that may increase the risk of relapse and ways to be attentive toward these factors
Session 10	Psychoeducation about good advice to avoid binge-eating. The patient is able to read the goals they set in Session 2. Exercise: The patient is asked to write a farewell letter to their eating disorder

The treatment program has been proven effective in treating BED symptomology (30).

### Measures

Based on the DSM-5 diagnostic criteria, we developed the Binge-Eating Disorder Questionnaire (BED-Q) to screen for BED severity. BED-Q comprises 9 items. Item 1–7 construct the sum score from 0 to 35, interpreted as: 0 = no symptoms; 1–9 = subclinical symptoms of BED; 10–14 = mild BED; 15–21 = moderate BED; 22–28 = severe BED; and 29–35 = extremely severe BED. The items assess the frequency of seven BED symptoms, rated as follows: 0: none; 1: <1/week; 2: 1–3/week; 3: 4–7/week; 4: 8–13/week; and 5: >13/week. Item 8 controls for compensatory behaviors with response categories similar to items 1–7. Item 9 assesses whether binges are experienced as distressing with a binary yes/no. A Cronbach's Alpha analysis of 201 BED patients showed that the seven BED symptom items had an internal consistency of α = 0.79.

The Major Depression Inventory (MDI) (31) was used to screen for depressive symptoms. The MDI consists of 10 items rated on a 6-point Likert scale 0–5. The total score ranges from 0 to 50; the higher the score, the more symptoms of depression. Cut-off points are recommended at 21 for mild depression, 26 for moderate depression, and 31 for severe depression.

Quality of life was measured using a Visual Analog Scale (VAS) from the EuroQol-5 Domain 5 Level (EQ-5D-5L) (32). The patients rate their health from “the worst health you can imagine” (0) to “the best health you can imagine” (100) on a sliding scale. Sociodemographic variables included sex, age, and BMI.

### Qualitative data and analyses

In the present study, qualitative data consisted of written texts entered by the patients into the iBED treatment program. The texts addressed the patients' goals for their treatment course and their motives for seeking online treatment. The sample texts were collected from the treatment program, gathered in new documents, and applied identification numbers. The first data evaluation was blinded with regard to the distribution of completers and non-completers.

The written texts from the treatment program were analyzed qualitatively through of *systematic text condensation (STC)* (33). *STC* is a pragmatic, explorative, and descriptive method for thematic cross-case analysis of qualitative data which is well-suited for written texts. The qualitative analysis was conducted by authors TTH, ESJ and KT. According to *STC*, the analytic procedure consists of the following steps: *(1) Total impression—from chaos to themes*. During this first step, we established an overview of the data by reading the texts, and acquiring a general impression of the replies, while searching for preliminary themes. *(2) Identifying and sorting meaning units—from themes to codes*. At this step, we identified and organized the impressions created by the data to further elucidate the research question by systematically reviewing the texts for meaningful components known as units. *(3) Condensation—from code to meaning*. This step entailed systematic abstraction of units by reducing empirical data into thematic code groups, who were no longer attached to the body of text, but free floating construction blocks for the next step. *(4) Synthesizing—from condensation to descriptions and concepts*. At this stage, we synthesized the condensate contents into new meaningful emergences to help reveal facets of the research question.

Consensus on themes for motivation was achieved by authors TTH, ESJ, and KT, each designating one or more theme per patient statement. These were then compared, and in case of doubt, agreement was reached from discussion. Saturation was achieved by an inductive process by categorizing each patient's response and then comparing and discovering commonalities. Firstly, we determined all themes for each patient. Secondly, we determined a main motivational theme for each patient. This was determined based on which theme the patient mentioned first and which theme they elaborated on the most. These condensates were combined with quantitative findings to examine preliminary hypothesis and further hypotheses generation. After emergence of themes, data was revisited based on the knowledge gained and further investigated based on the self-determination theory framework.

The raters were generally congruent and only a few patients were debated until consensus was achieved.

### Quantitative data and analyses

In conformity with the consensual and evidence-based belief in the impact of motivation on treatment effectiveness and outcome, all patient variables in the dataset were grouped by motivation type, and various statistical tests were conducted for each grouped variable. This was done in order to assess the significance of such groupings, identify significant patterns between groups, and more generally gain a better understanding of the relationship of each patient variable to motivation type.

Given that the different motivation types form an ordered sequence as per the self-determination theory, Jonckeheere-Terpstra tests (*T*_*JT*_) were conducted to look for ordered differences between motivation type-based groups for patient variables. The sign (+/−) of the test statistic T indicates *H*0: *Group*_1_ = *Group*_2_ = …*Group*_*n*_: positive scores are indicative of an ascending order between groups *H*1_*Asc*._:*Group*_1_ ≤ *Group*_2_ ≤ …*Group*_*n*_, while negative scores are associated with the alternative hypothesis of a descending order between groups, *H*1_*Desc*._:*Group*_1_ ≥ *Group*_2_ ≥ …*Group*_*n*_; in both cases, at least one strict inequality between groups is postulated (i.e., < for *H*1_*Asc*._ and > for *H*1_*Desc*._). As with other stochastic dominance tests, shifts in distribution location between the different groups are assessed based on mean ranks, but comparisons can also extend to group medians if the difference groups being compared are of similar variance. In light of this, Brown-Forsythe homoscedasticity tests (*F*_*BF*_) were also conducted on each variable grouping in order to determine the precise nature of the partial orderings detected.

Regarding effect size, given that the variables considered in this study are either ordinal or non-normally distributed, strength of association between each analyzed variable pairing was assessed using Kendall's tau-b (τ_*B*_) estimations (the τ_*B*_ statistic is used due to the presence of ties in the data), along with 95% confidence intervals. As is the case with most correlation coefficients, τ_*B*_ can be conceptually interpreted as indicating proportional reduction in prediction error (34), which means that it can also be used to evaluate effect size between variables in terms of the magnitude of shared variance between them in a manner similar to Pearson's coefficient of determination (*r*^2^) (35, 36).

In the case of pre/post measures, we used a factorial repeated measures ANOVA test. We chose this test despite non-normality, because ANOVAs are in general robust to violation of the normality assumption, and because our sample is large enough that the sampling distribution approximate the shape of a normal distribution (37).

Finally, as regards to the various significance thresholds adopted in this study, *p*-value-based tests will be considered significant if *p* ≤ 0.05, and significance levels will be represented symbolically alongside each result based on the standard asterisk rating system. In other words, results from the Jonckheere-Terpstra test will be considered significant if *p* (T_*JT*_) ≤0.05, and distributions will be considered of unequal variance if *p* (F_*B*_*F*) ≤0.05; the same reasoning also applies to all factorial ANOVA results. Regarding effect size, the recommended minimal practical effect size (RMPE) of *RMPE* = |0.2| for strength of association indices is used as threshold value, along with recommended values for moderate (|0.5|) and strong (|0.8|) effects (36); results meeting these increasing thresholds will be assigned one to three asterisks, based on the strength of association. With this in mind, strength of association results deemed significant have to satisfy two conditions. First, based on the chosen threshold value, the point estimate obtained from the test statistic (τ_*R*_) has to lie outside of the [−0.2,0.2] interval. Additionally and in order to keep the probability of an association being inversely significant (significantly negative instead of significantly positive as reported by the point estimate or vice versa) below 5%, lower and upper bounds of the corresponding 95% confidence interval cannot both reach the significance threshold, namely −0.2 for the lower bound and 0.2 for the upper bound. Finally, for both the Jonckheere-Tersptra and Kendall's tau results, only cases that prove significance both in terms of stochastic dominance and effect size will be considered.

### Sentiment analysis

Sentiment analysis can be defined as the computational identification, extraction, quantification, and analysis of linguistic expressions of affective states and subjectivity (38–40). Despite this general definition, however, most research and algorithms developed in the field of natural language processing focus on polarity detection, which aims to represent the emotional content of linguistic entities as scores on a scale from strongly negative to strongly positive (40–50).

Now a fundamental tool of computational text analysis, polarity detection (henceforth polarity) has notably been used in psychology for analyzing sentiment in dreams (51), detecting mental disorders in Tweets (52), diagnosing anorexia nervosa (53, 54), and assessing the wellbeing of Tweeters during the pandemic (55).

For the current project, polarity of the patient's answers to both motivational questions was extracted using a modified version of the Sentida package (56, 57), a hybrid (both lexicon- and rule-based) polarity detection algorithm for Danish-written texts. Summarily, Sentida extracts polarity from text in a three-step process: by means of a lexicon, scores ranging from −5 (negative) to 5 (positive) are first assigned to words, adjusted through rules accounting for non-interrogative negations, exclamations, and modifiers on perceived sentiment value, and then aggregated at the sentence or document level through either sum or arithmetic mean pooling. Given the proven shortcomings of the two score aggregation techniques used by the algorithm (58), a general form of geometric averaging, more robust and designed to process both neutral and negative values (59, 60), has been implemented and applied to each patient's answer regarding their therapy aims and their perception of online therapy.

### Ethics

In accordance with Danish national ethical guidelines, no ethical clearance was sought for the present sub-study, as the research was solely based on questionnaire data from the iBED treatment study. The iBED study is conducted in accordance with the ethical standards of the institutional and national research committee and with the 1964 Helsinki Declaration and its later amendments or comparable ethical standards. All patients gave digital written informed consent for their treatment data to be used for research purposes. Regional Committees on Health Research Ethics for Southern Denmark approved the iBED study (20212000-57). The study was reported to the Danish Data Protection Agency.

## Results

### Descriptives

BMI for the sample at the beginning of the treatment was 37.5 [21.5- mean age 39.6 (18–68)], 17 out of 148 were male (11.5%), mean depression score of 23 (2–42). Nineteen out of 148 where young adults. Completion rates can be viewed in Table 2.

**Table 2:** Overview of completion rate frequencies by age group.

**Age group**	**Completion rate**	**Frequency**	**% Age group**
Adult	Full	66	25.78
	High	98	38.28
	Low	92	35.94
Young adult	Full	8	21.05
	High	14	36.84
	Low	16	42.11

### Patient motivations for seeking BED treatment

The systematic text condensation of the written texts led to the construction of themes, entailing descriptions of patient motivations for seeking BED treatment. Four main themes, which reached a saturated level in the sample, were discovered: avoidance of guilt/shame, desire for tools/insights, weight loss, and psychological stress. Patients ranged from one emergent theme to four. In the patients describing more than one theme, a primary motivation was determined based on how early it was mentioned, and how many words the patient used to describe each theme.

We then coded the data into which type of motivation each group displayed. In this sample, amotivation as per the model from Ryan and Deci (20), would not appear to be present as all patients have had the agency to act by signing up for treatment. Purely intrinsic motivation is seemingly not present, as no patient describe wanting treatment because it is a fun, enjoyable process. They all have an outside goal to achieve, which they hope the treatment will help them reach, therefore are all motivated extrinsically in varying degrees, from introjection, identification to integration. We defined statements as more autonomously driven, if they were exemplified by self-reference, by the patients taking responsibility, i.e., a more internalized locus of causality. An example of an autonomous motivational statement is: “*I hope that via the psychological tools, that can help people like me with BED, I can have a natural relation with food and not use it as a surrogate for the real problem”* (patient 45). An example of a more controlled motivational statement is: “*I am so sad about my situation. I'm ashamed and do not want my girls to have the same relationship with food as I do. I can't control it alone and there is no one around me who understands*” (patient 147).

#### Avoidance of guilt and shame

One motivation for seeking treatment was to get rid of feelings of guilt and shame: “*I often eat so much I feel physical discomfort and afterward I experience sadness and am repulsed”* (patient 8). Shame is a controlled type of motivation and that is also often the case in this sample. “*My overweight and the shame that comes with it. Both when shopping, eating and gatherings*” (patient 28). These patients fear the social stigma of being overweight, they self-shame and wish for treatment to escape this, indicating focus on approval from themselves and others. Therefore, shame is placed in the second least internalized and somewhat externally regulated category, introjection, controlled. This also means that the perceived locus of causality is mainly external, meaning that the patients in this category perceive low agency.

#### Weight loss

Losing weight was also a concern for a large part of the patients. Patients described having problems with being overweight influencing them negatively or directly stating that they wanted to lose weight: “*I have hope about changing my eating habits and thereby achieving weight loss*” (patient 39). Regarding weight loss, many of the patients who desire weight loss suffer from physical ailments worsened by their BED. They wish for better physical health, which can be perceived as more autonomous than introjection, as they are working for themselves and not the perception of others. There is, however, mainly focus on weight loss and how it will improve their lives, not so much on how they wish to achieve it, and generally agency is not described. A typical example from this group: “*I have for many years eaten as BED… I pushed it away and have not taken it seriously. But a couple of years ago, I had back problems and this winter I was diagnosed with yet another health problem…. I am not that overweight… Now I need surgery and was told the surgeon only operates on people with a normal BMI…. Which means I have to lose xx kilos*” (patient 41, potentially identifying health data changed by authors to a similar situation). These patients are more motivated by some external situation, such as needing surgery. However. this patient for example, does not perceive them self as that overweight despite having a BMI of 29, which indicates less identification and less internalized locus of causality, wherefore, this group appears to perceive agency as more external than internal locus of causality and we classify it in introjection.

#### Psychological stress

Psychological stress was the most reported problem that motivated people to seek treatment. Patients were motivated by eliminating psychological stressors from everyday life: “*I spend a lot of unwanted energy on it every day and it drains me”* (patient 51). The psychological stress group appear to have a more internalized locus of causality than the two previous ones. The patients want to feel better about themselves, and express agency, but at the same time, there is still avoidance present in this motivational type. They are mainly reaching out for help to avoid negative emotional distress. “*I want help to control my food intake and avoid using food to feel better. I am tired of always having to think about what I eat*” (patient 189). These patients reach out for help, to feel better. They are still not fully engaging in taking accountability, indicating a not fully internalized locus of causality. Therefore, we label this group as in the identification subset of extrinsic motivation.

#### Wish for insight and obtaining self-help tools

Table 3 shows how patient motivations were distributed in the “therapy aims” setting. Table 4 shows how the motivational types were distributed on age group. Another motivation for seeking help was getting to know themselves better and/or acquiring specific tools to help themselves: “*I hope to acquire tools and an insight that will enable me to control my food intake*” (patient 22). Thus, the insights category is more autonomous than the previous ones. The patients who focus on acquiring tools and insight into their disorder, as they are focusing on how they specifically can achieve agency, and often with and understanding of potential underlying causes “*I am reaching out because I need help to get a hold of my BED and my food patterns and where everything is coming from. Food takes an incredible amount of space and I want to down-prioritize it, using the right tools*” (patient 186). Here, the patient acknowledges a lack of tools, a sense of prioritizing and an understanding that it comes from somewhere within themselves.

**Table 3:** Distribution of patients' motivational types regarding therapy aims.

***Controlled→ Autonomous***					
**Motivational type**	**Introjection**	**Introjection**	**Identification**	**Integration**	
Patient motivation:	Shame	Weight loss	Psychological stress	Insight	In all
In all	25	25	50	48	148

**Table 4:** Distribution of motivation type, by motivational question and age groups.

**Motivational question**	**Age group**	**Motivation type**	**Frequency**
Therapy aims	Adult	External	0
		Introjection	47
		Identification	42
		Integration	39
	Young adult	External	0
		Introjection	3
		Identification	8
		Integration	8
Why online therapy	Adult	External	24
		Introjection	19
		Identification	71
		Integration	14
	Young adult	External	7
		Introjection	2
		Identification	9
		Integration	1

### Patient motivations for seeking online treatment

The systematic text condensation of the written texts led to the construction of themes, entailing descriptions of patient motivations for seeking online treatment. Four main motivations that reached a saturated level in the sample were discovered: external recommendation, avoidance of stigma, convenience, and reflection on treatment course. As with the patient motivations for seeking BED treatment, we also divided the patient motivations for seeking online treatment into motivational types.

#### External recommendation

The external recommendation group had experienced someone telling, suggesting, or recommending them to seek online treatment for BED. If there had been other possibilities, they might had chosen differently. An example of this is patient 20: “*It was recommended to me. I called Odense Hospital on a number I found in an article about BED. They told me this was the best fit for me.”* External regulation is recognized by compliance and reactance (20). An example of the patients' reactance and compliance came from patient 63 who simply stated: “*Since this is what was recommended to me*”.

#### Avoidance of stigma

The avoidance group liked the non-stigmatizing and anonymous character of online treatment. The choice of online treatment was impacted by feeling ashamed of their condition and embarrassed by sharing. One patient elaborated: “*I read about the treatment and it immediately caught my interest as it is hard to talk about because many people just think that I need to pull myself together and stop overeating*” (patient 25). Thus, they found it easier to hide behind the screen and formulate themselves in writing making them feel less like “patients” suffering from a condition. One patient explained it like this: “*Positively (…) it makes me more anonymous which I think is nice as this is a difficult subject for me to talk about”* (patient 34). The avoidance group was categorized as introjection type motivation, as it did have some ego involvement, but was focused on approval from self and others as seen in patient 12 who states “*I am too embarrassed to tell anyone about it”*.

#### Convenience

The largest group was the convenience group, consisting of patients who felt that online treatment seems more familiar and easier to work with, enables staying at home, eliminates transport time, as well as enables receiving help when suitable, and can be attended to besides work and family. Here, this flexibility is described by patient 9: “*If the help is offered during unfortunate times in relation to my other chores, it can sometimes feel more as a burden than a help. Therefore, the internet-based treatment is attractive as I can work with my eating disorder when it is relevant for me. At the same time, the treatment is situated when I have time, opportunity and surplus. I find this liberating and it makes me want the help even more because it does not become a stress-factor. At the same time, I can reserve all the time I need for treatment when I decide when it is situated. And I am convinced that it requires time*.” The convenience group, we categorized as identification, exactly because the patients expressed this treatment type as being convenient for them, which indicates conscious valuing of activity and personal importance. Another representative example is from patient 76: “*Internet-based treatment is appealing to me, as I can do it from home, and make it fit into my everyday life”*.

#### Reflection on treatment course

The reflection group mentioned the fact that the internet-based solution offers the possibility to have a quiet and peaceful treatment course as having an impact on them choosing online treatment. One patient expressed it like this: “*I think it will be good for me to follow treatment in my own environment at home, and that I can read feedback, do the exercises, and what else the treatment entails when I have the peace to do so”* (patient 90). The peace and quiet was emphasized as lucrative as it enabled sitting alone, reflecting on the questions in the treatment program. One patient expressed it like this: “*The elasticity in the treatment form makes it easier for me to immerse myself in the treatment when I have the peace and quiet to think and act”* (patient 0029). In addition, it was emphasized that the writing could provide an overview. Reflection was categorized as integration motivation type. This was based on patients not only wanting online treatment because it was convenient, but preferring the qualities it entailed explicitly, such as seen with patient 26: “*Because I sit and read and write a lot on the computer, and I think that will make it easier for me. And also because it is only in my home I eat sugar!”* Our synthesis generated Table 5.

**Table 5:** Distribution of patients' motivational types regarding online treatment.

***Controlled→ Autonomous***					
**Motivational type:**	**External**	**Introjection**	**Identification**	**Integration**	
Patient motivation:	External	Avoidance	Convenience	Reflection	In all
In all	31	21	81	15	148

When comparing the motivation types of adults and young adults there was too little data to run a test. However, when viewing the descriptives from Table 5, there is not obvious difference between the two groups.

We then compared motivational content for both “*therapy aims*,” and “*why online*” on treatment outcomes and adherence.

### Statistical analyses

Before undertaking any analyses, normality was assessed by generating detrended quantile-quantile plots (61) for the different variable value distributions, followed by Shapiro-Wilk normality tests on each variable. All variable distributions were significantly non-normal.

#### Motivation type

Table 6 shows the results for the stochastic dominance and effect size tests. Jonckheere-Terpstra analyses showed that the only variable whose test results are significant both in terms of stochastic dominance and effect size is polarity score. This is the case both for the patient group as a whole for adult patients. In both cases, the motivation group distributions also proved to be of similar variance, which means that the ordered sequence observed for the different group mean ranks also applies to group medians, with at least one strict order (<) between group pairs.

**Table 6:** Statistical interaction between motivational type and patient variables, by motivational question and age group.

			**Stochastic dominance**		**Homoscedasticity**		**Effect size**	
**Variable**	**Motivational question**	**Age group**	***T*_*JT*_**	***p*(*T*_*JT*_)**	***F*_*BF*_**	***p*(*F*_*BF*_)**	**τ_*B*_**	***CI*_95*%*_**
Sentiment polarity score	Therapy aims	All	5.12	3.0e−07***	0.08	0.93	0.31*	(0.20, 0.42)
		Adult	4.85	1.3e−06***	0.21	0.81	0.32*	(0.21, 0.44)
	Why online therapy	All	3.77	1.6e−04***	0.31	0.73	0.27*	(0.14, 0.40)
		Adult	3.54	4.1e−04***	0.28	0.76	0.27*	(0.13, 0.41)
		Young	1.92	0.05*	0.90	0.44	0.48*	(0.12, 0.84)
BEDQ (Pre)	Why online therapy	All	−2.15	0.03*	0.54	0.58	−0.17	(−0.32, −0.01)
		Adult	−2.22	0.03*	0.42	0.66	−0.18	(−0.35, −0.02)
BMI (Pre)	Why online therapy	Adult	−2.01	0.04*	4.14	0.02*	−0.16	(−0.33, 0.01)

Only cases with significant stochastic dominance results, as given by the Jonckheere-Terpstra test statistic (*T*_*JT*_) and its associated *p*-value, are shown here. For each of the latter, statistics and *p*-values from the Brown-Forsythe homoscedasticity test (*F*_*BF*_) are also included, along with effect size results obtained using Kendall's tau (τ_*B*_) with a 95% confidence interval (*CI*_95%_). BEDQ, Binge Eating Disorder Questionnaire; BMI, Body Mass Index.

**Figure 1:**
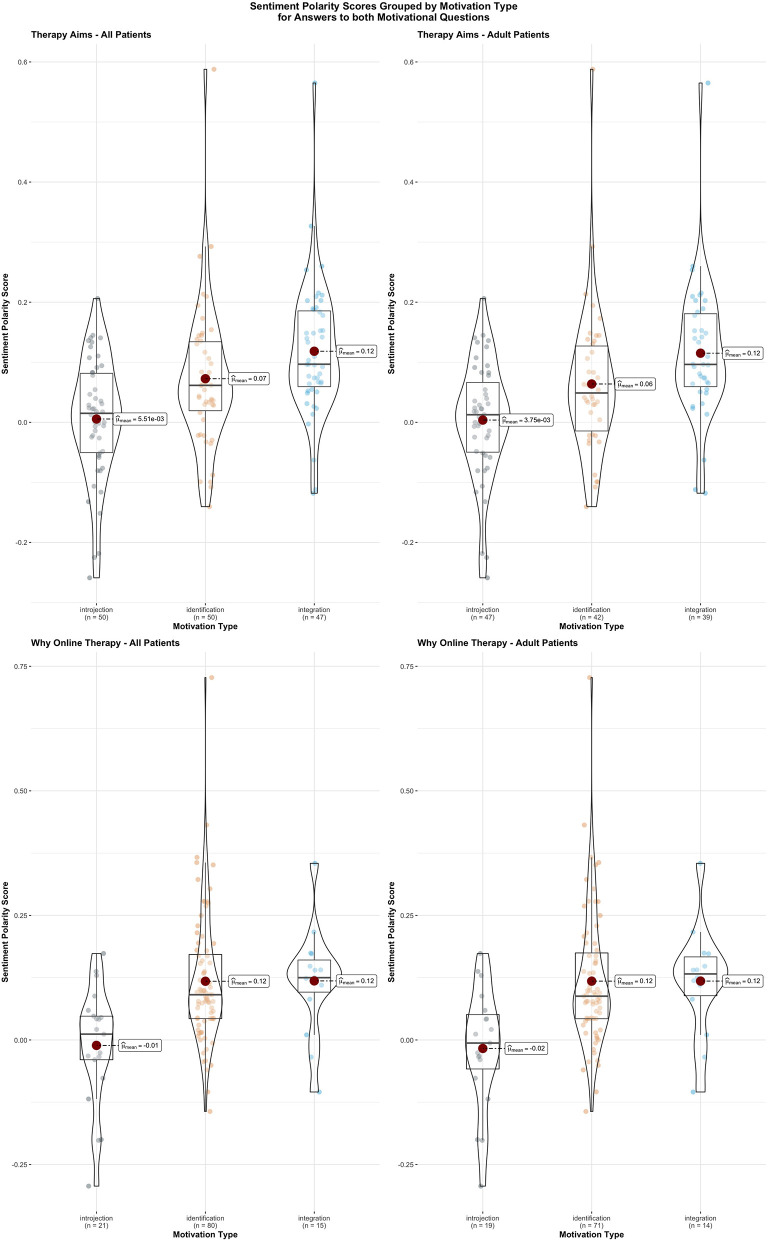
The correlation scores between sentiment polarity and motivation type, grouped by motivational question and age group. Groups means as well as box and whisker plots are added for each violin plot.

Figure 1 demonstrates the significant effect of the correlation between sentiment polarity and motivation type. It only contains adult and all patients, as the young adult group does not include any significant results, even though a visual trend was visible.

In terms of effect size, τ_*B*_ scores in the abovementioned cases are all over the recommended minimum threshold; in the case of sentiment polarity scores for all patients' and adult patients' answers regarding therapy aims, even the lower bounds of both confidence intervals are significant in that same aspect.

To sum up, the above results indicate a statistically significant association between increases in sentiment polarity scores and progression in motivation types for answers to both motivational questions. Regarding effect size, results indicate that a significant amount of the variance shared between both variables can be explained by variation in motivation type. Given this, it can be concluded that polarity scores constitute an efficient proxy for motivation type.

There is less clarity of the young adult group regarding whether the polarity score correlates with motivation type. It is less significant, but the effect size is large, so if there is a bigger sample, significance might occur.

#### Sentiment polarity

To better understand the interaction between motivation-related emotional content and both treatment effectiveness and outcome, τ_*B*_ correlation scores between sentiment polarity and completion rate as well as both binge eating disorder score differentials were collected for the whole patient group and both age groups respectively. As can be seen in Table 7 below, the only significant correlation score here refers to the relationship between the polarity score of young patients answers as to why they chose online therapy and completion rate: the more positive the score, the more they are likely to complete the program.

**Table 7:** Correlations between seq13ntiment score and treatment outcome, by variable type and motivational question.

		**All patients**		**Adult**		**Young adult**	
**Variable type**	**Motivational question**	**τ_*B*_**	***CI*_95*%*_**	**τ_*B*_**	***CI*_95*%*_**	**τ_*B*_**	***CI*_95*%*_**
BEDQ T1	Therapy aims	0.01	(−0.10, 0.13)	−0.02	(−0.14, 0.11)	0.12	(−0.29, 0.53)
	Why online therapy	0.01	(−0.10, 0.12)	9.1e−04	(−0.12, 0.12)	0.06	(−0.29, 0.41)
EDEQ T1	Therapy aims	−0.03	(−0.14, 0.08)	−0.05	(−0.16, 0.06)	0.15	(−0.25, 0.56)
	Why online therapy	−0.05	(−0.15, 0.05)	−0.04	(−0.14, 0.06)	−0.15	(−0.57, 0.27)
Completion rate	Therapy aims	−1.4e−03	(−0.13, 0.13)	−4.1e−03	(−0.15, 0.14)	−0.03	(−0.35, 0.30)
	Why online therapy	0.13	(3.5e−03, 0.26)	0.18	(0.05, 0.32)	−0.31*	(−0.66, 0.04)

BEDQ, Binge Eating disorder Questionnaire; EDEQ, Eating Disorder Examination Questionnaire.

#### Treatment outcome

The factorial repeated measures ANOVA demonstrated no significant effect of motivation type on treatment outcome (EDEQ, BEDQ, MDI, VAS) in the “*therapy aims*” setting.

There was no significant effect of motivation type on treatment adherence as seen in Table 8.

**Table 8:** Results of factorial ANOVA tests on the interaction between motivational types and various patient variables.

**Motivational question**	**Outcome from T1 to T2**	**numDF**	**denDF**	***F*-value**	***P*-value**
Therapy aims	BEDQ	2	774	0.08	0.92
	EDEQ	2	774	0.68	0.51
	MDI	2	774	0.02	0.98
	VAS	2	774	0.20	0.82
Why online therapy	BEDQ	2	618	5.4e−03	0.99
	EDEQ	2	618	1.48	0.23
	MDI	2	618	4.4e−03	1.00
	VAS	2	618	0.12	0.88

BEDQ, Binge Eating Disorder Questionnaire; EDEQ, Eating Disorder Examination Questionnaire; MDI, Major Depression Inventory; VAS, Visual Analog Scale; numDF, degrees of freedom numerator; denDF, degrees of freedom denominator.

## Discussion

### Motivational factors

Based on the literature, we expected a correlation between adherence and motivation types, which we did not find. There was no significant correlation between how integrated the patients motivations were, and how likely they were to complete the program. Moreover, there was no correlation between how much effect they gained from the program and their motivation type.

In addition, the fact that the population has very narrow motivational types was unexpected. At first, we thought that internet-based therapy automatically sorts out the most externally, controlled motivations because it requires an amount of agency, and populations without it, may not even apply. However, we did find the complete lack of even a single patient with external regulation motivation in the “*therapy aims*” a bit odd, so we contacted the psychologists, who screened the patients for the program, and found that it was an exclusion criterion for them. This meant that if a patient wrote “*there is nothing else available*” or a similar statement, they would not be included in the program. As we talked about this, it became clear that this was not a conscious decision from the therapists in the sense that they thought that the patients were externally motivated and would not be as fit for the program. This decision was based on a clinical sense the clinicians developed *via* previous experience with face-to-face therapy with this population. The screeners have worked with over 200 BED patients and have more than 20 years of combined experience in ED, so it is safe to assume a well-developed clinical sense. This is of course a confounding variable. They did not use the same screening exclusion for the online motivation criteria, which is why external motivation is present in that sample.

Therefore, we checked to see if external regulation motivation in the “*why online*,” had any influence on treatment adherence or effect, compared to the other motivation types.

There was no significant difference from other types, so externally regulated patients in the “*why online*” were as likely to adhere to treatment as more autonomously motivated types. This could indicate that external regulation is not as disadvantageous to treatment adherence in online treatment as it is in face-to-face treatment, which would make sense. Motivation influences adherence, because when you go to an appointment in person, you first have to take time off from work/family or other obligations, which also means telling your boss or significant other, where you are going and why. You have to face potential fear and shame, which are very prevalent in this population, and overcome them each time you go there. Being in therapy on its own can also be an unpleasant zone of proximal development experience, where you challenge yourself and face less flattering facts about yourself and your situation. Each of these potentially negative experiences opens up to potential avoidance behavior/avoidance coping strategies being triggered, which could be particularly hard for this population to overcome because as the avoidance theory suggests (12, 62) the patients are trying to escape self-awareness, *via* their overeating.

In the program, no one sees you—not even your therapist. You can perform the treatment when it is convenient, and you can even hide the fact that you are in treatment, so it diminishes the perceived positive effect of avoidance behavior. This could mean the more externally regulated patients should not necessarily be excluded from the program.

However, we will have to include them in the future to find out for sure.

Because of the confounder of the screening process, we cannot say much about external regulation motivation in the “*therapy aims*” and the effect it would have on treatment.

A study on an online self-help program for anorexia nervosa by Monteleone et al. investigated if motivation could be affected *via* motivational interviewing as part of treatment and if that would influence treatment. They found no such effect (63). This collaborates the disconnect found between motivation type and treatment outcome. However, the current study and Monteleone et al. (63) are not directly comparable, and future studies will have to investigate further.

Another possible reason for the findings could be that shame is an especially powerful drive in patients with BED. We see high levels of shame in the patients and that could explain why motivation type does not predict adherence and outcome as most literature suggests (22–25).

Because of this, this finding should be replicated in other patient groups outside of eating disorders.

### Optimizing the program

As the program is CBT-based, it could be relevant to look into the motivations as to give an indication if other treatment methods could be incorporated, such as Acceptance and Commitment Therapy (ACT) or pure behavioral training. Having these traits of the population in mind when expanding and designing treatment programs is prudent, as addressing avoidance, enhancing acceptance of guilt/shame, reframing weight loss focus, and alleviating psychological stress, even before treatment onset, could benefit this population (64, 65).

### Online treatment factors

External factors had an impact on 31 patients choosing online treatment for BED. It was somewhat surprising that these patients did not have a worse outcome than patients who chose the program because it was their preference, hinting at the fact that patients perception of online treatment, may not affect treatment adherence. Concerning the themes for seeking online treatment, we found four major themes of importance for seeking internet-based treatment.

1) Avoidance of stigma had an impact on patients choosing online treatment for BED. We found that their choice of online treatment was specifically impacted by the fact that the treatment could be conducted in their own home. Important factors were not wanting to leave the house due to being introverted or suffering from anxiety, and the desire to remain anonymous during the treatment due to feeling ashamed and embarrassed. In a systematic review assessing treatment barriers in EDs, shame/stigma is mentioned as the first barrier found across the 11 reviewed studies (64). This indicates that online treatment may be a barrier breaker for ED patients when it comes to seeking treatment.
2) Furthermore, convenience also had an impact on patients choosing online treatment for BED. We also found that they chose online treatment due to a preference for receiving treatment *via* the internet. Important factors were receiving help for self-help and the effectiveness of online treatment. This finding is in agreement with previous studies, which have indicated that internet-based treatment may be one way to increase access to treatment for mental health disorders (66, 67). According to recent reviews, guided-self-help CBT is a promising evidence-based intervention which can assist in reaching those who are in need but unlikely to get treatment (68).
3) Moreover, we found that the patients' choosing online treatment was influenced by the flexibility of treatment. Here, important factors were living far away from treatment opportunities and that the treatment could comply with an otherwise busy everyday life with work and family. Again, this finding is in line with the review on treatment delivery strategies for EDs (68) which also found that mobile-health approaches had the potential to overcome geographical barriers.
4) Finally, we saw that having the possibility to calmly reflect during treatment was an essential aspect of choosing online treatment. This finding is consistent with findings from a review on treatment delivery strategies for EDs about mobile-health approaches having the potential to overcome personal barriers, and increase reach by allowing patients to access the interventions anonymously, when and where they want, and at their own pace (68). This final focal point demonstrates where online therapy has an advantage compared to face-to-face treatment. You can reread every section as many times, as you want, and if you need a break to think, you can do that, and then continue at your own pace.

Overall, the patients describe many of the advantages of online therapy. This underlines that online treatment is not necessarily directly comparable or in competition with face-to-face treatment, as it can do something face-to-face cannot. Moreover, face-to-face has effects that online treatment does not. They have the potential to be complimentary.

Another thing to note is something that is not mentioned in reasons for choosing online therapy. In a systematic review assessing treatment barriers in EDs, cost of treatment is mentioned as a barrier found across the 11 reviewed studies (64). The iBED program is free of charge and often in studies where treatment is free, people will note cost as a factor for seeking the treatment (65). Three patients in this setting mentioned this. Perhaps because the general lack of treatment options for this patient group made the sample more economically diverse. It is also possible that the lack of focus on cost is because the other motivational themes were more important for the patients.

### Sentiment polarity and motivation type

The directional correlation between sentiment polarity and motivation types is a very interesting find, which we did not anticipate to the degree it was found. The fact that the higher the polarity the more autonomous the motivation was, demonstrated that we in future studies, might be able to use the algorithm for text analysis instead of manually scoring them. This will save time and resources.

It takes time and requires specific expertise to code 148 patient statements. Using an algorithm still requires knowledge, but most support staff can do it.

Being able to determine motivation type quickly may lead to new discoveries in research, and can potentially help patients in the future, as we may be able to detect changes in motivation over type *via* written feedback from patients, or we can better understand which motivation types are most suited for online treatment.

As for the model of the correlation between sentiment score and motivation type, this finding validates the validity of the coding, as we did not know that patients were excluded based on this, when we coded the data. If the coding criteria were faulty, the chance of us falsely denoting statements as external motivation would be large.

Therefore, we can also more surely say that the correlation between sentiment score and motivation type is significant and based on well-coded data.

The “*why online*” had lower polarity score than “*therapy aims*,” indicating lower emotional involvement. This indicates that the patients were not that positive or negative regarding having to do online therapy.

The model was not as strong when looking at the young adult group as the sample was smaller, however, there was an negative association between sentiment polarity and adherence in the “*why online*” setting. This could be because the younger group has a different set of expectations for online therapy than the older group. Increasing the risk of them being overly expectant and positive about online therapy, which may not be what they imagined. If this is the case, this could explain why the association is negative, however; further studies and a bigger sample is needed to investigate this difference.

### Strengths and limitations

The majority of patients were female, and less is known about males' reasons for seeking BED-treatment. However, since BED is the most prevalent ED in males (69), it will be prudent to investigate this subsample on its own and compare it with the female population. This would increase knowledge about how BED manifests in men and how treatment differs. In future studies, the relationship between controlled and autonomous motivation in an online setting, should be further investigated, to discern if motivation type in this group is less significant for treatment adherence. To further test whether motivation influences outcome measures, employing the Autonomous and Controlled Motivation for Treatment Questionnaire (ACMTQ) in future samples as well as in a larger sample size would be beneficial.

The volume of the text-base differed from patient to patient, increasing the risk of type II error, as some patients may not have stated all their reasons.

Therapeutic alliance is also a confounding variable in treatment adherence. The general contact during the program between therapist and patient, was out of scope for this paper, but should be included in future studies, to illuminate this possible confounder/mediator. It seems especially important if the motivation type is more external (25).

The lack of external regulation motivation in the “*therapy aims*” sample is both a strength and a limitation. We do not know how this motivation type would have influenced data, since they were excluded before treatment. However, we now know that data was coded true to the self-determination theory, as we uncovered this limitation because of the lack of patients with external regulation as motivation in the sample.

A strength of this paper is the mixed methods design. Using the patients' qualitative statements to inform and enhance the quantitative measures, gives us insights that would have been hard to gain *via* either strategy alone.

## Conclusions

Motivation types in regards to therapy aims found in the sample were limited to the more or less internalized types, from introjection, and integration to internalization. Amotivation and purely intrinsic types were not found nor was external regulation found in the “*therapy aims*” setting. Regarding the “*why online*” setting motivation types included these three, but also external regulation. However, the screening process was partially the reason for this picture. The qualitative findings indicate that the online treatment offer iBED may be able to breach some of the barriers toward treatment seeking such as convenience, avoidance of shame and increased ability to reflect. Results also indicate that motivation types are not determinant factors for adherence or treatment outcome in an online setting for treating BED. This holds true for both adults and young adults. Perhaps a change in the perception of clinical screening of patients receiving online therapy is needed. Motivation types that are usually disadvantageous for treatment, may be less so online and online therapy may be a tool that can enable more extrinsically motivated patients to receive a treatment, which they can adhere to. Sentiment was positively correlated with motivation type, this means that sentiment analysis of written text maybe developed as a tool to help inexperienced or non-clinicians to assess motivation type of patients, and/or follow patients treatment and intervene if motivation changes. The treatment is equally effective in the younger population and they do not differ significantly in motivation type, so a future adaptation of the program to a younger target demographic would be plausible.

## Data availability statement

The data that support the findings of this study are available on request from the corresponding author. The data are not publicly available due to privacy or ethical restrictions.

## Ethics statement

Ethical review and approval was not required for the study on human participants in accordance with the local legislation and institutional requirements. The patients/participants provided their written informed consent to participate in this study. Written informed consent was obtained from the individual(s) for the publication of any potentially identifiable images or data included in this article.

## Author contributions

TH, KT, and EJ coded the data. TH and KT coded the themes and did the analysis. TH and MS-M wrote main text of the manuscript. JL and ML developed the treatment program, gave feedback, and supervision. ER helped with literature search and discussion. MS-M performed the sentiment analysis and statistical analysis. All authors contributed to the article and approved the submitted version.

## Funding

The mental health services in the region of Southern Denmark has funded the project and the open access fee.

## Conflict of interest

The authors declare that the research was conducted in the absence of any commercial or financial relationships that could be construed as a potential conflict of interest.

## Publisher's note

All claims expressed in this article are solely those of the authors and do not necessarily represent those of their affiliated organizations, or those of the publisher, the editors and the reviewers. Any product that may be evaluated in this article, or claim that may be made by its manufacturer, is not guaranteed or endorsed by the publisher.
